# Regional Cross-Sectional Based Study and Associated Risk Factors of Porcine Circovirus 2 in Nigerian Pigs

**DOI:** 10.1155/2023/9201177

**Published:** 2023-12-16

**Authors:** Kayode O. Afolabi, Olufemi S. Amoo, Tochukwu I. Onuigbo, Joy I. Oraegbu, Ayomikun A. Awoseyi, Folorunso O. Fasina, Oluwawemimo O. Adebowale

**Affiliations:** ^1^Department of Microbiology and Biochemistry, University of the Free State, South Africa; ^2^Molecular Epidemiology and Public Health Research Group (MEPHREG), Department of Biological Sciences, Anchor University, Lagos, Nigeria; ^3^Centre for Human Virology and Genomics Research, Nigerian Institute of Medical Research NIMR, Lagos, Nigeria; ^4^Computer Sciences Department, First Technical University, Ibadan, Oyo, Nigeria; ^5^Food and Agriculture Organization of the United Nations, Rome, Italy; ^6^Department of Veterinary Tropical Diseases, University of Pretoria, Pretoria 0110, South Africa; ^7^Department of Veterinary Public Health and Preventive Medicine, Federal University of Agriculture Abeokuta, Abeokuta, Ogun State, Nigeria

## Abstract

Porcine circovirus 2 (PCV2) is a swine viral pathogen of substantial economic importance in pig farming globally. However, large-scale surveillance is needed to determine its prevalence and associated risk factors in the Nigerian pigs. We conducted molecular-based surveillance and mapping of PCV2 in southwest Nigeria to assess its prevalence and spatiality. Six hundred forty-eight individual fecal samples were collected from the different age groups of pigs from 67 farms in three southwest states. The polymerase chain reaction technique was used to screen the samples with a specific primer pair. The viral prevalence was determined at individual animal and farm levels. Overall, 145 out of 648 samples (22.4%) and 49/67 farms (73.1%) tested positive for PCV2. The highest prevalence of PCV2 was observed in Oyo State (63/185, 34.1%) and in growers (66/145, 45.5%). Restricting visitors' entrance to the farm was found to be strongly protective for PCV2 (AOR 0.122; *p*=0.007; 95% CI; 0.027–0.564), while not having a quarantine protocol (AOR 4.445; *p*=0.041; 95% CI; 1.067–18.5280) and reporting coccidiosis as a common disease encountered (AOR 14.340; *p*=0.007; 95% CI; 2.094–98.203) on the farm were significant risk factors identified to be associated with the presence of PCV2. This study revealed a higher prevalence of PCV2 in Nigerian swine herds than expected and presented significant spatial clustering of infection in the studied region. It has also highlighted the risk factors driving its spread in the studied area. The research findings underscore the need for a policy decision to promote PCV2 vaccination in the country, which is currently not in place. The availability and use of the PCV2 vaccines, in addition to effective biosecurity measures, will help to mitigate the virus and its associated diseases in the country for sustainable and profitable pig farming, which holds vast potential in solving the problem of hunger and poverty.

## 1. Introduction

Porcine circoviruses (PCVs) are single-stranded circular DNA viruses with an icosahedral naked capsid of 12–23 nanometers in size [1, 2]. The smallest known animal viruses belong to the genus *Circovirus* in the family *Circoviridae* alongside the cycloviruses [3, 4]. Three types of PCVs are present in the pigs, including porcine circovirus types 1, 2, and 3 (PCV1, PCV2, and PCV3); however, a novel PCV has been discovered recently and designated PCV4 [5]. The very first swine circovirus, namely PCV1, was detected by Tischer et al. [1, 6] as a contaminant of the pig kidney cell line (PK-15) but was proven to be nonpathogenic to swine [7].

Notably, PCV2, discovered in the late 1990s, changed the narratives about swine circoviruses due to its pathogenicity. The viral pathogen is associated with the porcine multisystemic wasting syndrome (PMWS) and other porcine circovirus-associated diseases (PCVADs), which include porcine dermatitis and nephropathy syndrome (PDNS), enteritis, proliferative and necrotizing pneumonia (PNP), porcine respiratory disease complex (PRDC), and reproductive failure [8]. Preferentially, PCV2 targets the lymphoid tissues when an animal is infected, leading to lymphoid depletion and suppression of immunity in pigs. The infection is further worsened by immunostimulation or coinfection of PCV2 with other pathogens, leading to many disease outcomes [9]. Although PCV2 has many transmission routes, the major routes include oro-nasal contact with feces and urine from infected animals or through direct contact. The virus is readily shed in body secretions and feces of clinically and subclinically affected animals [10]. It has been reported to be detectable as early as 1-day postinoculation (dpi) and could persist for at least 70 days, as shown in the experimental infection studies [11–13].

Presently, nine genotypes of PCV2 exist, namely PCV2a to PCV2i, though only three of the genotypes (PCV2a, PCV2b, and PCV2d) are globally distributed with significant swine health consequences [14]. Initially, the PCV2a was the predominant genogroup till around 2002/2003 when a form of “genotypic shift” was observed, perhaps due to the worldwide use of PCV2 vaccines, leading to the predominance of PCV2b. Subsequently, another genotypic shift occurred in 2010; PCV2d is presently becoming the predominant genotype in pigs globally [15, 16].

PCV2 is a swine pathogen of substantial economic importance, associated with myriads of diseases capable of decimating productivity in pig farming. For example, the economic loss incurred in England due to PMWS alone, one of the numerous PCVADs, in some farrow-to-finish facilities prior to the large-scale vaccination implemented in 2008, was put at £52.6 million and £88 million per year during the endemic years and when there was an outbreak, respectively [17]. This depicts a typical debilitating economic loss that could ensue due to PCV2 infection if not curbed. Although there is availability of data to show that PCV2 is a pathogen with devastating economic impacts globally, investigation into the existence and prevalence of the virus is still lacking in many sub-Saharan African countries [18].

For instance, the virus was detected in South African pigs, even on commercial farms with a high level of biosecurity by Afolabi et al. [19] when they made an appreciable effort to investigate its occurrence and prevalence following its first case report in the country [20]. The findings from the group's molecular surveillance helped bridge the information gap earlier identified by Mokoele et al. [21] about the lack of specific surveillance to validate the prevalence of PCV2 and its associated diseases in South African pigs. Undoubtedly, it is evident that the research landscape on the virus has relatively improved in the African region in recent years. Many reports have been made about its prevalence in some other countries, such as Uganda [22, 23], Mozambique [24, 25], South Africa [26], and Namibia [27]. However, it is glaring that much is still needed, as the currently available data on PCV2 prevalence in the region need ramping up.

Nigeria is one of Africa's top three pig-producing countries, with a population of over 7.5-million pigs [28]. However, the level of risk communication and community engagement (RCCE) to improve knowledge, education, and awareness of PCV2 among pig farmers in the country needs improvement. In a cross-sectional survey across two southwestern states, 79.2% (89/111) of farmers interviewed were unaware of PCV2 [29]. It is, however, noteworthy that some recent studies have confirmed the presence of PCV2 in the Nigerian pigs [30–32], but those studies were limited regarding sample size and area of study coverage. These authors have recommended large-scale epidemiological surveys to understand its national prevalence.

Southwest Nigeria has some of the densest pig populations in Nigeria, and understanding the epidemiology of PCV2 in this region will give a good overview of the national situation. We, therefore, present the first region-wide molecular-based surveillance of PCV2, its spatiality, and associated risk factors in Southwest Nigeria. Data generated from the study should provide a comprehensive picture of the virus prevalence and distribution in Southwest Nigeria and the associated predictors for its presence. It should also give scientific evidence for developing and implementing informed mitigation programs or policies against the viral pathogen.

## 2. Materials and Methods

### 2.1. Study Location

**Figure 1:**
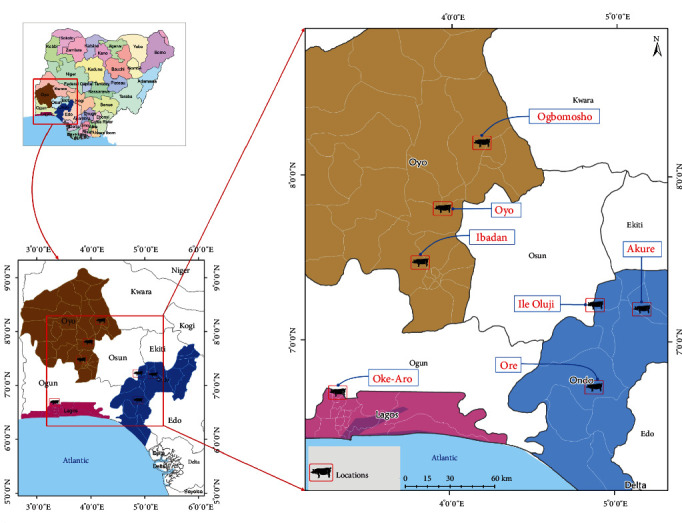
Geospatial presentation of study locations investigated for PCV2.

Nigeria is a federal constitutional republic in West Africa between latitudes 4–14° N and longitudes 2–16° E. It occupies a land area of 917,156 km^2^ and consists of six geopolitical zones (region), 36 states, and one federal capital territory (FCT) [33]. The study was conducted in three randomly selected southwestern states of Nigeria, namely Oyo, Lagos, and Ondo States, using the balloting technique (Figure 1). The study region was purposively selected, being Nigeria's highest pig-producing geopolitical zone.

### 2.2. Study Design and Sample Size Calculation

A regional-based cross-sectional study involving the administration of a semistructured questionnaire to farm owners/managers and the collection of fecal samples from pigs in Oyo, Lagos, and Ondo states, Nigeria, was conducted from December 2021 to February 2022. The sample size for this study with an estimated pig population in the region (https://www.statista.com/statistics/1297919/stock-of-live-pigs-in-nigeria/) was estimated using Epi Info 7 and the formula and assumptions: *n* = (*Z*2*P*(1−*P*))/*d*2 where: *n* is the required sample size; *Z* is the multiplier from a standard normal distribution (1.96) at a probability level of 0.05; *P* is the estimated prevalence using the default of 50%, assuming that there is no prevalence data of the disease or virus in Nigeria, and *d* is the desired precision for the estimate (+/−4%). A minimum of 601 pigs was estimated. Overall, 648 samples were collected for this research.

#### 2.2.1. Ethical Considerations and Informed Consent

Prior to the recruitment of participants for the study, the ethical approval to conduct the research was obtained from the College of Veterinary Medicine Research Ethics Committee (CREC), the Federal University of Agriculture Abeokuta (FUNAAB), Ogun State, Nigeria, with reference number FUNAAB/COLVET/CREC/2021/09/01. Also, written permission was obtained and signed by the chairpersons of the selected states' Pig Farmers Association of Nigeria (PFAN). Informed consent was verbally acquired from participants and witnessed by the association's state chapter chairpersons.

#### 2.2.2. Questionnaire Design and Pretest

The questionnaire has 53 questions comprising both open and closed-ended options. It was written in English and divided into four sections (1–4). The first section consisted of questions assessing the sociodemographic profiles of the respondents. These included age as at last birthday (in years), gender (M/F), marital status (married/single/divorced), highest educational level (no formal education/primary/secondary/tertiary), and primary occupation. To determine the farm and pig herd characteristics, the second section comprised six questions intended to gather information on the following: location of the farm (local government area (LGA); longitude/latitude), years of establishment of the farm, herd number and age, pig breeds, number of enclosures or pens, and the surface type of the enclosure.

Section three of the questionnaire assessed farm management and biosecurity. The section comprised of Yes/No and Likert scale, an approach to rate responses as regards biosecurity practice measures—item (34) questions that investigated farming and production systems, housing and feeding, biosecurity practices (4 Likert-item—Always/Frequently/Rarely/Never), awareness about PCV2 (Yes/No), and waste disposal methods. The score for each response was summed up to give a maximum of 92 points. The last section gathered necessary information on flock health status, antimicrobial use, and PCV2/postweaning multisystemic wasting syndrome awareness.

The questionnaire was reviewed for specificity (without ambiguity) and content validity by a panel of qualitative research experts from the Department of Veterinary Public Health and Preventive Medicine, FUNAAB. The tool was revised based on the comments and feedback, and it was pretested among 10 farms (excluded from the study) before the commencement of the field study. Further modifications were implemented based on the outcome of the pretesting.

#### 2.2.3. Sample Collection, Preparation, and Storage

A total of 648 fecal samples were collected from the commercial pig farms (*n* = 67) from the representative states. None of the farms recruited for the study carried out vaccination against PCV2 at the time of sampling based on our findings during questionnaire administration; most were unaware of the virus. In each farm, the samples were randomly collected from different age groups and categories of pigs and transported on ice packs from farms to the laboratory, processed using 1% phosphate buffer saline (PBS) and kept in a −80°C freezer until analyzed.

#### 2.2.4. Total Genomic DNA Extraction and Molecular Detection of PCV2

The molecular aspect of the study was carried out at the Centre for Human Virology and Genomics Research, Nigerian Institute of Medical Research (NIMR), Lagos. The total genomic DNA extraction and PCV2 detection were done according to Afolabi et al. [19] with slight modifications. In brief, the DNA extraction was done from the processed fecal samples using a NIMR genomic DNA extraction kit, with strict adherence to the manufacturer's procedures. Subsequently, the molecular screening of the samples for PCV2 was performed by polymerase chain reaction (PCR) using forward and reverse primers P1Fw (5′-TAATCCTTCCGAAGACGAGC-3′) and P1Rv (5′-CGATCACACAGTCTCAGTAG-3′) according to An et al. [34]. The primer pair targeted the 629-base pair (bp) long of the viral genome's open reading frame (ORF) 1 region (replicase gene).

The PCR mixture and conditions used for the molecular screening were according to the recommendation of the manufacturer of the master mix (Solis BioDyne) used, with some modifications. In brief, a 20-*µ*L PCR reaction mixture containing 4-*µ*L 5x Hot FirePol® Blend Master Mix, 0.4 *µ*L 10 *µ*M forward primer, 0.4 *µ*L of 10 *µ*M reverse primer, 0.4 *µ*L Magnesium chloride (MgCl_2_), 0.8 *µ*L dimethyl sulphoxide (DMSO), 5 *µ*L template DNA, and 9 *µ*L nuclease-free water, was used for the amplification process. The PCR conditions used for the process are as follows: 95°C initial denaturation for 15 min; 30 cycles of 95°C final denaturation for 20 s, 54°C annealing for 40 s and 72°C extension for 2 min; and 72°C final extension for 10 min. To further confirm the specificity of the primers used for the screening process, some selected PCV2-positive amplicons were sequenced, and the sequences obtained were processed, blasted, and submitted to the NCBI database. Phylogenetic analysis of the sequences was also done using MEGA 11 [35].

### 2.3. Statistical Analyses

Data generated were captured and filtered in Microsoft Excel, 2016 (Microsoft Corporation, Redmond, WA). Descriptive statistics were conducted for all categorical variables and presented in frequencies and proportions/percentages. For the numerical variables such as age, herd population, and respondents' scores for biosecurity level, the measures of central tendency (arithmetic mean and median), measures of variability (standard deviation), and absolute numbers (*n*) and percentage representation were generated [36]. The measured outcomes were tested for normality using the Kolmogorov–Smirnov (>0.05), which informed our use of Mean ± SD or median (minimum, maximum).

The farm biosecurity practice level of respondents was evaluated by giving scores of “1” for “never,” “2” for “rarely,” and 3 for “frequently,” and “4” for “always” responses, and 1 for “yes,” and 0 for “no” depending on the questions asked. The levels of measured score outcomes were expressed as mean and standard deviation (Mean ± SD). The scores were then converted to percentages, and the cutoff point was determined based on the average score of responses. Therefore, farms with scores > average cutoff were considered to have satisfactory biosecurity, while those ≤ average cutoff were considered to have poor biosecurity levels.

The farmers' demographic variables (age/gender/marital status/education/occupation) were tested for association with biosecurity level (poor/satisfactory). Risk factor analysis was conducted to determine predictors for PCV2 (absent/present), which included the farm/pig characteristics, management and biosecurity measures, and other transboundary animal diseases experienced by pig farmers, including African swine fever (ASF) and foot and mouth disease (FMD). Farm/pig characteristics, farm biosecurity measures and farm management were categorized into binary outcomes. Pearson's chi-square or Fisher's Exact tests (where appropriate) were determined (Table S1). Outcomes significant at *p* ≤ 0.25 at the univariate analysis were further processed by a backward stepwise likelihood multivariate analysis (logistic regression model) using SPSS 23.0 [37]. The decision for a liberal *p*-value (*p* ≤ 0.25) at the univariate step was to ensure important potential predictor/risk variables were included in the model. A *p* < 0.05 was considered statistically significant, and odds ratios were computed to determine the strength of associations between variables at 95% confidence intervals (CIs). The Omnibus test of model coefficients was used to provide the overall statistical significance of the model and to know how well the model predicts the categories. The Hosmer–Lemeshow test was used to assess the model's goodness fit.

For the geomapping, the hot spot areas of PCV2 are shown in color variations to indicate areas with the presence or absence of the pathogen within the pig farm boundaries. Areas of hot spots are shaded red, while those without the pathogen are green. Spatial autocorrelation (SA) was performed to know whether there was any cluttering, randomness, or dispersion in the porcine circovirus type 2 (PCV2) using “Spatial Autocorrelation (Moran's *I*),” which is a subset of “Spatial Statistics Tools” in ArcMap 10.5 [38]. Hotspot Analysis (Getis-Ord Gi ^*∗*^) was also conducted.

## 3. Results

### 3.1. Geospatial Distribution of Study Locations

The various sampling locations investigated for PCV2 are presented (Figure 1).

#### 3.1.1. Demographics of Pig Farmers and Farm Characteristics

Sixty-seven farms were visited, but 66 farmers completed the farm manager questionnaire. Most of the pig farmers were males (71.2%), married (92.4%), and had tertiary education (71.2%). The mean age of respondents was 43.8 years (SD ± 14.7), and 33.3% self-reported that pig farming was their primary occupation. Generally, 53/66 farms (80.3%) had herd size ≤100 pigs (median 48, min 1, and max 250). The most common breeds of pigs kept by respondents were the large white (86.4%), followed by Duroc (39.4%), Landrace (34.8%), and Yorkshire (9.1%) in descending list. Piglets, growers, weaners, sows, and boars were present on 63.6%, 80.3%, 43.9%, 3.0%, 87.9%, and 72.2% of farms, respectively.  Table 1 presents the mean age and numbers of the various pig categories on the investigated farms. The median number of pens was 15 (min 1, max 50), and 42.4% of farms had ≤15 enclosures on the farm. All (100.0%) farms had concrete flooring enclosures and at least one farm attendant.

#### 3.1.2. Farm Management and Biosecurity

Information gathered showed mainly pig production (92.2%) and intensive farming system (92.5%). Many respondents have quarantine protocol (73.1%) and practiced open farming (76.1%). All participants (100.0%) have never imported pigs from other countries. Pigs were fed with compounded and industrial feeds (94.0%) and crop residues (83.6%). Farms visited were ranked as having low (49.3%), moderate (40.2%), and high (10.5%) biosecurity by field surveyors. Lower proportions of farms observed cleaning and disinfection of farm premises (28.3%), enclosures (34.3%), and equipment (35.8%). The common waste disposal method was selling off wastes as fertilizers (65.7%), while the least practiced were burning/burying (29.9%) and open dumps (10.4%). The biosecurity level for all farms was a mean score of 47.9 ± 11.6, translating to a mean level of 52.1% (SD ± 12.5%, range 19.5%–80.4%). A total of 35 farms (53.0%) were classified as having poor biosecurity level ≤52.1% cutoff, with the lowest level being 19.5% (range 19.5%–52.1%). The rest had satisfactory levels, ranging from 52.2% to 80.4%.

#### 3.1.3. Farm Health Status and PCV2 Awareness

**Figure 2:**
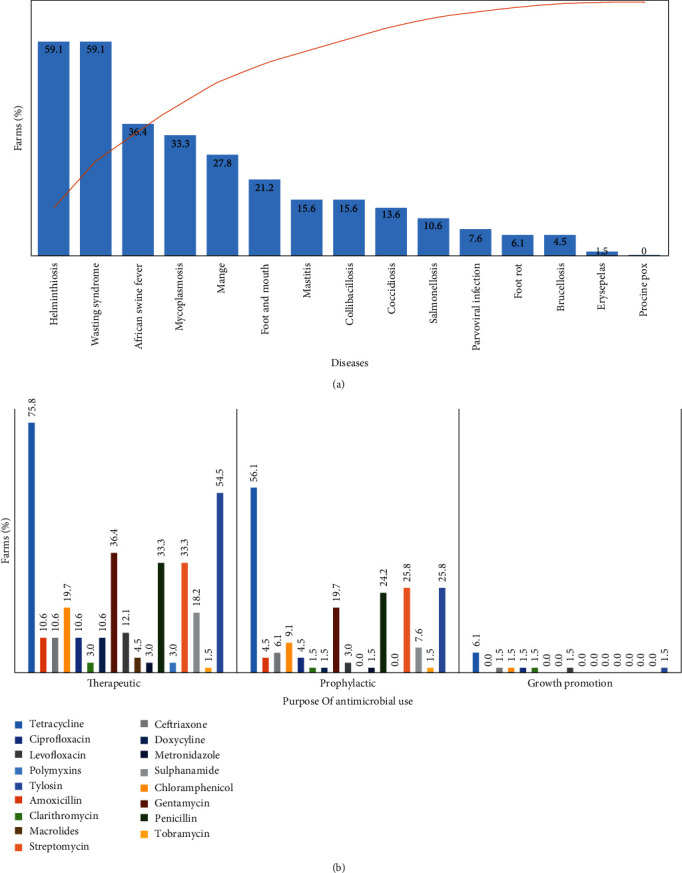
(a) Common diseases encountered among pigs and (b) antimicrobial use pattern among pig farms in Southwest Nigeria.

For the farm health status, the common diseases encountered, veterinary consultation, and antimicrobial usage were assessed.  Figure 2(a) presents the common diseases encountered on pig farms in the Southwest Nigeria. Helminthiasis and wasting disease syndrome were the most common (59.1%). All the farms (100.0%) routinely use anthelmintics (deworming agents) as prophylaxis or therapeutic, while 15.2% and 1.5% use probiotics and vaccines, respectively. Lower numbers of farmers (10.6%) consult veterinarians. For antimicrobial use, 95.5% of respondents used antimicrobials on the farms. Antimicrobials were used mainly for therapeutic and prophylactic purposes. Common antimicrobials used in descending list were as follows: tetracyclines (93.9%), tylosin (60.6%), penicillin–streptomycin (47.0%), and gentamycin (45.5%). Clarithromycin, metronidazole, polymyxins, and tobramycin were indicated as being used for growth promotion (Figure 2(b)).

Poor awareness about PCV2 was observed among pig farmers in Southwest Nigeria. Only two participants (3.0%) indicated being aware of PCV2 and have observed suspected PCV2 infection in piglets. Some clinical signs identified by these farmers as being associated with the disease included diarrhea, lack of appetite, poor and stunted growth, and stillbirth.

#### 3.1.4. Farm and Animal-Level Prevalence of PCV2 in Southwest Nigeria

**Figure 3:**
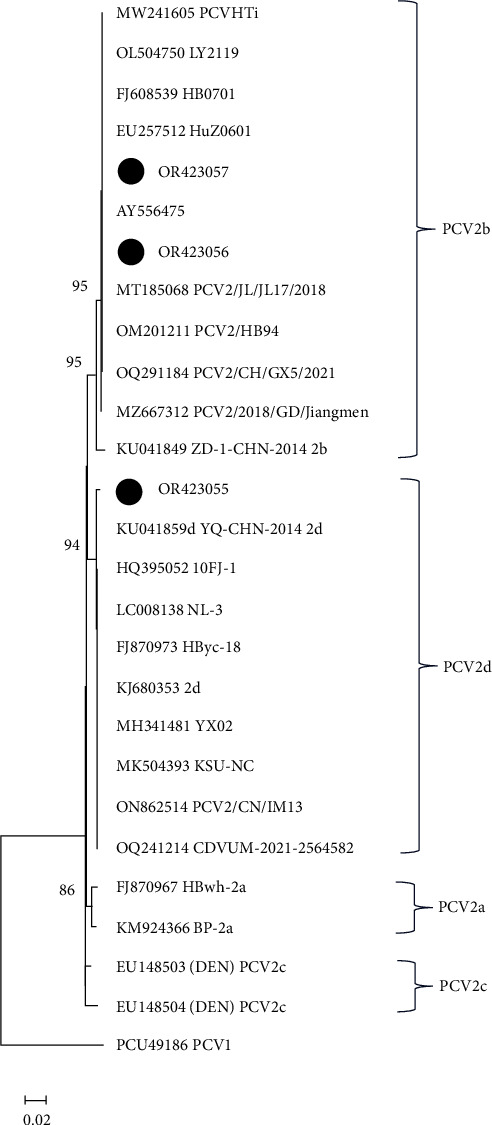
Phylogenetic tree showing three PCV2 sequences (in black dots) generated from the molecular screening exercise and the related reference sequences obtained from NCBI GenBank. The neighbour-joining method was used, and bootstrap values ≥80% are shown. A reference PCV1 (PCU49186) was used as an outlier.

The overall regional farm-level and animal-level prevalence obtained for PCV2 were 73.1% and 22.4%, respectively. At the state level, the farm prevalence rates were 93.3% (14/15), 70.0% (14/20), and 63.6% (21/32), while animal level prevalence was 34.1% (63/185), 20.1% (40/199), and 15.9% (42/264) in Oyo, Lagos, and Ondo states, respectively. PCV2 was most detected among growers (45.5%), followed by weaners (24.1%) and sows (22.8%). However, the lowest detection of PCV2 was in boars (7.6%). PCV2 was higher among growers (50.8%) and sows (27.0%) in Oyo State; growers (45.2%) and weaners (35.7%) in Ondo State; and growers (37.5%), weaners (27.5%), and sows (27.5%) in Lagos State (Table 2). The BALST result of the three sequences obtained from the screening exercise showed similarity ranging from 99.4% to 100% with other reference PCV2 sequences in the GenBank. The sequences were submitted to GenBank with the Accession numbers: OR423055, OR423056, and OR423057.  Figure 3 shows the clustering pattern of the three sequenced partial ORF1 genomes of the PCV2 obtained in this study, with two clustering with PCV2b and one with other reference PCV2d sequences.

#### 3.1.5. Mapping of the PCV2 Hot Spots in Southwest Nigeria

**Figure 4:**
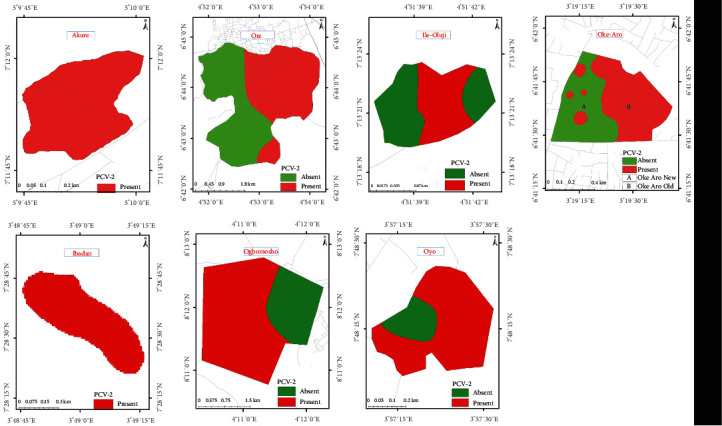
Hot spots (color-coded red) for PCV-2 in the sampled areas of Southwest Nigeria.

**Figure 5:**
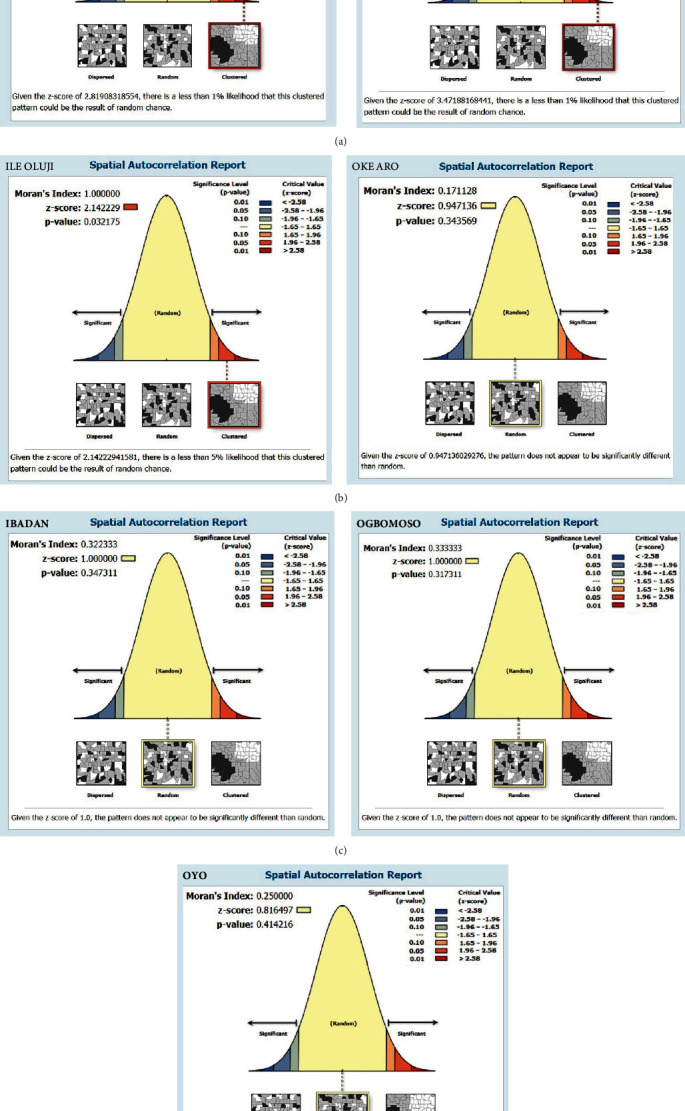
(a–d) Autocorrelation analysis of sampling sites for PCV2 in Southwest Nigeria.

The hot spot areas and spatial autocorrelation of PCV2 among farms investigated in Southwest Nigeria are presented in Figures 4 and 5, respectively. The results revealed a positive spatial autocorrelation (Moran's *I* = 0.361, *p* < 0.001), indicating a significant clustering pattern. Areas with high-PCV2 prevalence were spatially associated with neighboring areas of high prevalence, while areas with low prevalence also exhibited clustering.

Specifically, the analysis of Ore pig farms demonstrated interesting geospatial patterns related to PCV2 levels. The Moran's *I* value of 1.00 indicated a strong positive spatial autocorrelation, suggesting that farms with similar PCV2 levels were clustered together in the area. This finding supported the notion of localized disease prevalence. The low *p*-value of 0.0005 indicated that the observed spatial clustering was statistically significant, further confirming the presence of a spatial pattern. Additionally, the high *z*-score of 3.47188 indicated that the observed Moran's *I* value significantly deviated from what would be expected under the null hypothesis of spatial randomness. Similarly, at Ile-Oluji, analysis revealed a Moran's *I* value of 1.00, indicating a strong positive spatial autocorrelation. This suggested that pig farms with similar PCV2 levels were clustered together in the area. The low *p*-value of 0.03 indicated the statistical significance of the observed spatial pattern, suggesting that the clustering of PCV2 levels in Ile-Oluji was unlikely to occur randomly. The *z*-score of 2.142 further supported the significance of the spatial clustering pattern, indicating a statistically significant positive spatial autocorrelation.

In contrast, for Oke-Aro in Lagos State, Moran's *I* value of 0.171128 indicated a less pronounced spatial autocorrelation pattern than Ore and Ile-Oluji (in Ondo State). This suggested that the PCV2 levels in Oke Aro may exhibit less clustering or similarity among neighboring farms. The *p*-value of 0.343 indicated that the observed spatial pattern was not significantly different from spatial randomness. The *z*-score of 0.947 suggested that the observed Moran's *I* value was close to what would be expected under spatial randomness.

### 3.2. Univariate Analyses: Pig Farmers' Sociodemographic Variables and Farm Biosecurity Level; Farm Characteristics, Biosecurity Measures, and Presence of PCV2 in Southwest Nigeria

For the association between farmers' sociodemographics and farm biosecurity level, there was a statistically significant association between gender and biosecurity level only, *χ*^2^ = 4.929, *p*=0.03 (Table 3). The details of the determined association between PCV2 and farm/pig characteristics, pig diseases experienced, biosecurity, and management measures are presented in Table S1. In brief, the farm and pig characteristics strongly associated with PCV2 included keeping Landrace (*χ*^2^ = 8.393, *p*=0.04) and having more than 15 enclosures (*χ*^2^ = 4.462, *p*=0.04). At the same time, feeding animals with hotel foods was found to be marginally associated (*χ*^2^ = 3.771, *p*=0.05). Porcine coccidiosis (*χ*^2^ = 4.203, *p*=0.04), as a common disease experienced, was significantly associated with PCV2, while others such as mange (*χ*^2^ = 3.259, *p*=0.07), colibacillosis and mastitis (*χ*^2^ = 3.069. *p*=0.08) were marginally associated. The use of probiotics for pigs was also associated with PCV2 (*χ*^2^ = 3.069, *p*=0.08). For the biosecurity, association with PCV2 was significant with visiting other farms (*χ*^2^ = 7.265, *p*=0.01) and having farm quarantine protocol (*χ*^2^ = 4.520, *p*=0.03), while the farm biosecurity level (*χ*^2^ = 3.660, *p*=0.06), restriction of visitors' access to the farm (*χ*^2^ = 3.771, *p*=0.05), and having cleaning and disinfection protocol (*χ*^2^ = 3.356, *p*=0.067) were marginally associated.

#### 3.2.1. Multivariate Analysis of Farm-Level Risk Factors for PCV2 in Southwest Nigeria

The potential predictors with *p*-value ≤ 0.25 were subjected to the multivariate model analysis. Although the univariate analysis generated several significant potential predictors for PCV2, the final multivariable logistic regression models resulted in limited significant risk and protective factors. Not having quarantine protocol increased the odds of PCV2 infection than in farms with quarantine protocol (AOR 4.445; *p*=0.041; 95% CI; 1.067–18.5280), and farms that have experienced coccidiosis are more likely to be infected with PCV2 than farms that have not experienced coccidiosis (AOR 14.340; *p*=0.007; 95% CI; 2.094–98.203). Restricting visitors' entrance to the farm was found to strongly reduce the odds of PCV2 infection (AOR 0.122; *p*=0.007; 95% CI; 0.027–0.564).  Table 4 presents the significant variables at *p* ≤ 0.05 in a multivariate logistic regression analysis.

## 4. Discussion

In this study, we investigated the prevalence of PCV2 and associated risk factors, contributing to the introduction and spread of PCV2 in swine herds, including those of the largest pig-producing region of Nigeria. The finding from the study has not only confirmed the presence of the viral pathogen in Nigeria, as reported by earlier authors, but has given a near accurate picture of its prevalence in Southwest Nigeria and, by implication, the country. Earlier, Aiki-Raji et al. [30] reported a seroprevalence of 1.4% for PCV2 in a slaughterhouse-based survey conducted in Ibadan, Oyo State, Nigeria. This prevalence is lower than that obtained for the same Ibadan city in our study (30.2%). The disparity could be explained in many ways: (1) it may be due to the low sensitivity of the serological method used for the surveillance study; (2) it could also result from increased incidence and spread of the virus over the last 5 years since the study was conducted; or (3) It may be linked to the survey locations—abattoir-based versus farm-based sampling. Since the time of this report, there have been no reported preventive and control measures in Nigeria to mitigate the spread of the virus [29].

Secondly, in a recent study by Eleazar et al. [31], the prevalence of PCV2 in Abeokuta, Ogun State, Nigeria, was 8.7% using the PCR method. The result is comparable to the one from Ile-Oluji in Ondo State (9.2%), one of the cities investigated in this study. Furthermore, Luka et al. [32] utilized 107 archived DNA extracted from multistate tissue samples (blood, spleen, liver, lung, and lymph nodes) from Nigeria, which were collected as part of a routine ASF monitoring program and screened for PCV2, PCV3, and porcine parvovirus 1 (PPV1). Notably, 28% of the samples were positive for PCV2 [32]. Although the number of samples analyzed in the study may be too small to give an accurate picture of the infection status of PCV2 in Nigerian pig herds [32], this prevalence indicated that PCV2 is an underappreciated challenge in Nigerian pig herds. Interestingly, this prevalence of 28% is comparable to the overall prevalence of 22.4% obtained in our region-wide study. More importantly, considering the farm-level prevalence of 73.1%, PCV2 may have spread far more than is known in the Nigerian swine herds, hence the call for an urgent effort by stakeholders to comprehensively evaluate and mitigate the proliferation of the viral pathogen. This becomes highly imperative due to the economic importance of the viral pathogen and its associated diseases, as previously highlighted [17].

A compelling surveillance study to determine the extent of spread and prevalence of any infectious pathogen and its associated diseases is the first step in the right direction to mitigate its ravaging effect, whether in humans or animals [39]. As it is presently, even though vaccines are available and are being used in many other pig-producing countries of the world to prevent and control PCV2 infections for optimal productivity, a country-wide vaccination program against the pathogen is lacking in sub-Saharan African countries, including Nigeria [18, 29], except in a few countries in Southern Africa. The paucity of data on the prevalence of the viral pathogen in the region may be contributing to the observed inaction of stakeholders in employing mitigating strategies. However, it is noted that the research landscape on PCV2 surveillance has improved in recent years in the sub-Saharan Africa region, with prevalence findings emerging from some countries, including Uganda, Mozambique, and Namibia.

In a seroprevalence study on 522 pig sera screened by Dione et al. [22] to determine the prevalence of bacterial and viral pathogens in smallholder pig systems in Uganda, PCV2 came second (50.8%) only to *Streptococcus suis* (73.0%). The value far exceeded what is obtainable in our current study. Similarly, Wilfred et al. [23] conducted a cross-sectional survey to determine the occurrence of PCV2 and its associated systemic disease (PCV2-SD) among pigs. They evaluated the level of awareness of stakeholders on PCV2 in Central Uganda. None of the farmers and only 16.7% of the animal health workers interviewed previously heard about PCV2-SD, and 25% of the pigs sampled, have implicating lesions of PCV2-SD, and were confirmed positive for PCV2 using immunohistochemistry and PCR assays. In another recent study in the same Uganda, the coinfection of PCV2 with other respiratory pathogens and gastrointestinal parasites was confirmed in smallholder pig production systems; hence, the authors called for improved biosecurity in pig production, to achieve a reduced pathogens incidence in herds [40].

In Southern Africa, a study covering nine districts of southern Mozambique reported a herd and farm-level PCV2 prevalence of 54% and 78%, respectively, using the PCR method [24]. In addition, PCV3 has been detected as a co-infection with ASF in pigs in Mozambique, possibly for the first time in Africa, with a prevalence of 7.5% [25]. In Namibia, a study by Molini et al. [27] has also reported the occurrence of PCV2 for the first time in the country, especially in the wild pigs, with a prevalence of 28.3% and 23.8% in commercial pigs and warthogs, respectively. This same study reported the presence of PCV2c for the first time in Africa.

Despite the increase in the available data about PCV2 prevalence in Nigeria and other countries in the sub-Saharan Africa region, the perception and level of awareness of farmers and other stakeholders in the pig industry about the pathogen are low and need some urgent intervention. In an earlier cross-sectional survey in the same study locations as the current study, 79.2% of pig farmers were unaware of PCV2 [29]. The finding aligns with the earlier observations of Wilfred et al. [23] in Uganda, where all farmers (100%) and 83.3% of animal health workers interviewed in a study have never heard about PCV2-SD. This poor awareness indicates that more effort is required to sensitize stakeholders and increase knowledge of the risk factors of spreading the virus. The need for specific risk factors analyses and knowledge of the drivers and practices in the pig value chain, that could enhance the spread of PCV2 must addressed [10]. Targeted farmers' education will ensure safe farming practices (SFP), including vaccination, that could help prevent PCV2 and many other swine infectious agents from infecting their farms.

In our risk factor analysis, some biosecurity measures, including restricting visitors' entrance into the farm and having a quarantine protocol, are significantly associated with PCV2. While prevention of free and uncontrolled visitors' access to farms confers strong protection to the farms that practice it, failure to have quarantine protocol poses significant risks. It was strongly associated with PCV2 infection in the sampled farms. The practice of implementing highest level of biosecurity in livestock farming, particularly the intensive production system, is the secret of successful pig farming [41, 42]. Although pig production has been identified as one of the most profitable livestock ventures, it has been associated with myriads of infectious diseases. Most of the diseases can decimate herds and cause enormous economic loss, for example in cases of ASF, which could bring about a hundred per cent mortality in any affected farm [18, 43].

Though the mortality of PCV2 is relatively low compared to ASF, the impact of PCV2 infections, such as PWMS in swine herds, could be debilitating. This is because PCV2 could serve as a primary agent, making pigs susceptible to the other secondary infections due to its ability to impair the immune system of the affected pigs [10, 44, 45]. This could be the reason behind an incessant economic loss being experienced by farmers in the region due to the frequent occurrence of diseases such as ASF. While vaccination remains an effective way to prevent and control PCV2, the need for tight biosecurity measures in pig farming cannot be overemphasized. Biosecurity has been recommended to complement vaccination programs for PCV2 mitigation [46–48]. Based on our findings, there is a need to sensitize farmers on SFP in terms of strict biosecurity measures.

Based on risk analysis, the incidence of PCV2 in farms was significantly associated with other diseases/pathogens such as Coccidiosis (*p*=0.04) and less significantly associated with Mange (*p*=0.07), Mastitis (*p*=0.08), and Colibacillosis (*p*=0.08). As a pathogen that causes multifactorial diseases such as PMWS, PCV2 has been widely reported to coinfect with many other bacterial, viral, and protozoal pathogens, leading to the aggravation of the disease condition of pigs [49, 50]. Invariably, it has been asserted that the high application of antibiotics by farmers in the studied region could be linked to the farmers' efforts to prevent or treat other secondary infections due to PCV2 infection with zero knowledge about the primary underlying factor that enhances the susceptibility of the animals to the secondary agents [29, 51]. However, there is a need for a large-scale experimental study in the future, in addition to the one already established by Luka et al. [32], on the prevalence of PCV2 coinfections with other pathogens in Nigerian pigs for mitigation purposes. Also, further effort is required in the genetic characterization of the virus to determine the circulating genotypes in the region, for a better understanding of the virus epidemiology in the region and the country at large. This is imperative as the phylogenetic analysis of the three sequenced partial PCV2 genomes in this study has already shown the genetic diversity of the virus, even though the ORF1 region used is not the recommended gene region for the molecular characterization of the viral pathogen.

The geospatial analysis revealed the presence of significant spatial clustering of PCV2 infections in the studied region. Identifying clustered scenarios implies the influence of localized factors in the spread and prevalence of PCV2, such as proximity to infected farms, shared biosecurity practices, environmental conditions, or other local factors facilitating disease transmission. These findings contribute to a better understanding of the geospatial dynamics of PCV2 and can aid in targeted interventions and control strategies.

## 5. Conclusions

In conclusion, this study has successfully presented an accurate picture of PCV2 prevalence in the Southwest Nigerian pigs and determined some risk factors that enhance its transmission and have implications for Nigeria. These findings and many others in sub-Saharan Africa are expected to create more awareness about the viral pathogen and its associated diseases in the region. It is also anticipated that more concerted efforts will emerge from different countries in the region to control the virus through effective vaccination programs and formidable biosecurity measures.

## Figures and Tables

**Table 1:** Various pig categories, ages, and numbers.

Pig categories	Mean age in weeks	Standard deviation	Median number	Minimum	Maximum
Piglets	4.0	±3.1	10	4	80
Weaners	8.5	±3.9	12	2	150
Growers	16.6	±5.8	20	1	100
Boars	44.3	±24.7	2	1	19
Sows	58.01	±33.4	6	2	29

**Table 2:** Farm and animal level prevalence of PCV2 among pigs in Southwest Nigeria.

S/N	State/area	No. of farms sampled	No. of PCV2 positive farms (%)	No. of pigs sampled	No. of PCV2 positive pigs (%)	No. of PCV2 positive weaners (%)	No. of PCV2 positive growers (%)	No. of PCV2 positive sows (%)	No. of PCV2 positive boars (%)
1	ONDO	32	21 (63.6)	264	42 (15.9)	15 (35.7)	19 (45.2)	5 (11.9)	3 (7.1)
	Ore	10	5 (50.0)	81	11 (13.6)	5 (45.5)	6 (54.5)	0 (0.0)	0 (0.0)
	Ile-Oluji	10	5 (50.0)	98	9 (9.2)	7 (77.8)	2 (22.2)	0 (0.0)	0 (0.0)
	Akure	12	11 (91.7)	85	22 (25.9)	3 (13.6)	11 (50.0)	5 (22.7)	3 (13.6)

2	Oyo	15	14 (93.3)	185	63 (34.1)	9 (14.3)	32 (50.8)	17 (27.0)	5 (7.9)
	Ogbomoso	4	3 (75.5)	44	16 (36.4)	4 (25.0)	9 (56.3)	3 (18.8)	0 (0.0)
	Oyo town	5	5 (100.0)	55	21 (38.2)	3 (14.3)	4 (19.0)	9 (42.9)	5 (23.8)
	Ibadan	6	6 (100.0)	86	26 (30.2)	2 (7.7)	19 (73.1)	5 (19.2)	0 (0.0)

3	Lagos	20	14 (70.0)	199	40 (20.1)	11 (27.5)	15 (37.5)	11 (27.5)	3 (7.5)
	New Oke-Aro	10	5 (50.0)	109	13 (11.9)	5 (38.5)	5 (38.5)	1(7.7)	2 (15.4)
	Old Oke-Aro	10	9 (90.0)	90	27 (30.0)	6 (22.2)	10 (37.0)	10 (37.0)	1 (3.7)
	Total	67	49 (73.1)	648	145 (22.4)	35 (24.1)	66 (45.5)	33 (22.8)	11 (7.6)

**Table 3:** Univariate analysis for the association between farmers' demographics and farm biosecurity level.

Farmers' demographic information	Biosecurity level (%)		*p* Value at 95% CL
	Poor	Satisfactory	
Age (years)			
<45	57.1	42.9	0.74, *χ*^2^ = 0.109
>45	52.9	47.1	
Gender			
Male	61.7	38.3	0.03, *χ*^2^ = 4.929 ^*∗*^
Female	31.6	68.4	
Marital status			
Single	60	40	0.75, *χ*^2^ = 0.106
Married	52.5	47.5	
Education			
Primary	66.7	33.3	
Secondary	69.2	30.8	0.28, *χ*^2^ = 2.548
Tertiary	46.8	53.2	
Primary occupation			
Pig farming only	54.5	45.5	0.86, *χ*^2^ = 0.03
Others	52.3	47.7	

**Table 4:** Multivariate logistic regression analysis for the association between farm characteristics and biosecurity measures and the presence of PCV2.

Variable	Category	Adjusted odds ratio (AOR)	95% CI	*p* Value
Quarantine protocol	Yes	Ref	NA	
	No	4.445	1.067–18.528	0.041

Visitors restriction from farm	No	Ref	NA	
	Yes	0.122	0.027–0.564	0.007

Reporting colibacillosis on farm	No	Ref	NA	
	Yes	0.122	0.027–0.564	0.007

*Note*: Ref, reference; NA, not applicable.

## Data Availability

The sequences generated in this study have been submitted to the GenBank database with Accession numbers OR423055-OR423057.

## Supplementary Materials

Supplementary Materials Table S1: univariate analysis for association between farm characteristics, biosecurity measures, and presence of PCV2.
