# Corrigendum to “Regional Cross-Sectional Based Study and Associated Risk Factors of Porcine Circovirus 2 in Nigerian Pigs”

**DOI:** 10.1155/tbed/9890640

**Published:** 2025-08-11

K. O. Afolabi, O. S. Amoo, T. I. Onuigbo, et al., “Regional Cross-Sectional Based Study and Associated Risk Factors of Porcine Circovirus 2 in Nigerian Pigs,” *Transboundary and Emerging Diseases* 2023, no. 1 (2023): 1–15, https://doi.org/10.1155/2023/9201177.

In the article, there are errors in Table 4. Specifically, the “Yes” AOR and CI values given for the “Reporting colibacillosis on farm” variable are incorrect. Please see the correct Table 4 below:

We apologize for this error.

## Figures and Tables

**Table 4:** Multivariate logistic regression analysis for the association between farm characteristics and biosecurity measures and the presence of PCV2.

Variable	Category	Adjusted odds ratio (AOR)	95% CI	*p* Value
Quarantine protocol	Yes	Ref	NA	
	No	4.445	1.067–18.528	0.041

Visitors restriction from farm	No	Ref	NA	
	Yes	0.122	0.027–0.564	0.007

Reporting colibacillosis on farm	No	Ref	NA	
	Yes	14.430	2.094–98.203	0.007

