# Supplementary material for: Regional Cross-Sectional Based Study and Associated Risk Factors of Porcine Circovirus 2 in Nigerian Pigs
Source: Transbound Emerg Dis. 2023 Dec 16;2023:9201177. doi: 10.1155/2023/9201177 (PMC12017129; doi:10.1155/2023/9201177)
Supplement: Supplementary Materials — Table S1: univariate analysis for association between farm characteristics, biosecurity measures, and presence of PCV2. [file 9201177.f1.docx]

**Supplementary Table 1. Univariate analysis for association between farm characteristics, biosecurity measures and presence of PCV2.**

| **Variables** | **PCV2 (%)**  **Absent Present** | | **P value, χ2** |
| --- | --- | --- | --- |
| **Farm/Pig Characteristics and Management** | | | |
| Herd size (number)  <48  >48 | 38.1  19.0 | 61.9  81.0 | 0.172, χ2= 1.867* |
| Pig breed  Large White  Yes  No  Landrace  Yes  No  Duroc  Yes  No  Yorkshire  Yes  No | 31.6  0.0  8.7  40.0  38.5  21.6  33.3  28.1 | 68.4  100.0  91.3  60.0  61.5  78.4  66.7  71.9 | 0.272, χ2= 3.908  0.039, χ2 = 8.393*  0.339, χ2 = 3.362  0.740, χ2= 1.254 |
| Age by Category (weeks)  Piglets  <4  >4  Weaners  <9  >9  Growers  <17  >17  Sows  <58  >58  Boars  <44  >44 | 23.1  27.3  25.0  20.0  15.6  21.1  26.1  0.0  27.3  16.7 | 76.9  72.7  75.5  80.0  84.4  78.9  73.9  100.0  72.7  83.3 | 0.786, χ2 = 0.074  0.760, χ2 = 0.093  0.623, χ2 = 0.242  0.154, χ2 = 2.035*  0.384, χ2 = 0.759 |
| Number by Category  Piglets  <10  >10  Weaners  <12  >12  Growers  <20  >20  Sows  <6  >6  Boars  <2  >2 | 40.9  20.0  33.3  16.2  30.4  16.7  41.4  17.9  22.9  28.6 | 59.1  80.0  66.7  83.8  69.6  83.3  58.6  82.1  77.1  71.4 | 0.143, χ2 = 2.143*  0.338, χ2 = 2.168  0.502, χ2 = 0. .452  0.121, χ2 = 0. .452*  0.674, χ2 = 0.177 |
| Number of Pens  <15  >15 | 39.3  14.3 | 60.9  85.7 | 0.035, χ2 = 4.462* |
| Enclosure Surface  Concrete  Non-concrete | 27.0  33.3 | 73.0  66.7 | 0.809, χ2 = 0.058 |
| Housing System  Open  Closed | 30.9  9.1 | 69.1  90.9 | 0.138, χ2 = 2.200* |
| Hotel Feed  Yes  No | 42.9  20.0 | 57.1  80.0 | 0.052, χ2 = 3.771* |
| Crop Residues  Yes  No | 28.6  20.0 | 71.4  80.0 | 0.575, χ2 = 0.314 |
| Industrial Feed  Yes  No | 25.4  66.7 | 74.6  33.3 | 0.117, χ2 = 2.459* |
| **Pig diseases/conditions reported** | | | |
| ASF  Yes  No | 33.3  23.8 | 66.7  76.2 | 0.403, χ2 = 0.698 |
| Salmonellosis  Yes  No | 28.6  27.1 | 71.4  72.9 | 0.935, χ2 = 0.007 |
| Foot and Mouth  Yes  No | 14.3  30.8 | 85.7  69.2 | 0.219, χ2 = 1.511* |
| Coccidiosis  Yes  No | 55.6  22.8 | 44.4  77.2 | 0.040, χ2 = 4.203* |
| Mycoplasmosis  Yes  No | 27.3  27.3 | 72.7  72.7 | 1.000, χ2 = 0.000 |
| Helminthiasis  Yes  No | 25.6  29.6 | 74.4  70.4 | 0.721, χ2 = 0.128 |
| Mastitis  Yes  No | 50.0  23.2 | 50.0  76.8 | 0.080, χ2 = 3.069* |
| Wasting syndrome  Yes  No | 28.2  25.9 | 71.8  74.1 | 0.838, χ2 = 0.042 |
| Porcine Parvo  Yes  No | 40.0  26.2 | 60.0  73.8 | 0.506, χ2 = 0.442 |
| Mange  Yes  No | 11.1  33.3 | 88.9  66.7 | 0.071, χ2 = 3.259* |
| Colibacillosis  Yes  No | 50.0  23.2 | 50.0  76.8 | 0.080, χ2 = 3.069* |
| Foot Rot  Yes  No | 50.0  25.8 | 50.0  74.2 | 0.292, χ2 = 1.109 |
| Brucellosis  Yes  No | 66.7  25.4 | 33.3  74.6 | 0.117, χ2 = 2.459* |
| Use of Probiotics  Yes  No | 50.0  23.2 | 50.0  76.8 | 0.080, χ2 = 3.069* |
| **Biosecurity** | | | |
| Biosecurity level  Poor  Satisfactory | 37.1  16.1 | 62.9%  83.9 | 0.056, χ2 = 3.660* |
| Surveyors’ assessment on Biosecurity  High/Moderate  Low | 18.2  36.4 | 81.8  63.6 | 0.097, χ2 = 2.750* |
| Bird-animal Proof  Always/frequently  Rarely/never | 8.3  31.5 | 91.7  68.5 | 0.103, χ2 = 2.652* |
| Separate Enclosure for Weaner  Always/frequently  Rarely/never | 25.5  0.0 | 74.5  100.0 | 0.411, χ2 = 0.676 |
| Separate Enclosure for Growers  Always/frequently  Rarely/never | 24.6  0.0 | 75.4  100.0 | 0.259, χ2 = 1.275* |
| Separate Enclosure for Sows  Always/frequently  Rarely/never | 28.1  0.0 | 71.9  100.0 | 0.379, χ2 = 0.773 |
| Separate Enclosure for Boars  Always/frequently  Rarely/never | 25.0  0.0 | 75.0  100.0 | 0.321, χ2 = 0.983 |
| Feed properly stored/covered  Yes  No | 20.7  32.4 | 79.3  67.6 | 0.288, χ2 = 1.130 |
| Foot dip at pen entrance  Always/frequently  Rarely/never | 21.7  30.2 | 78.3  69.8 | 0.460, χ2 = 0.545 |
| Visit other farms  Always/frequently  Rarely/never | 42.4  87.5 | 57.6  12.5 | 0.007, χ2 = 7.265* |
| Borrow Equipment from other farms  Always/frequently  Rarely/never | 17.4  33.3 | 82.6  66.7 | 0.170, χ2 = 1.886* |
| Marketers/middlemen have unrestricted access to pigs  Always/frequently  Rarely/never | 27.7  23.5 | 72.3  76.5 | 0.741, χ2 = 0.109 |
| Vehicles allowed unrestricted access to the farm  Always/frequently  Rarely/never | 23.1  36.0 | 76.9  64.0 | 0.262, χ2 = 1.259 |
| Visitors have unrestricted access to farm  Always/frequently  Rarely/never | 21.4  37.5 | 78.6  62.5 | 0.158, χ2 = 1.989* |
| Visitors have restricted access to farm  Always/frequently  Rarely/never | 20.0  42.9 | 80.0  57.1 | 0.052, χ2 = 3.771* |
| PPE Provided to visitors before access to farm  Always/frequently  Rarely/never | 20.0  28.8 | 80.0  71.2 | 0.566, χ2 = 0.330 |
| All in –all out  Always/frequently  Rarely/never | 33.3  26.5 | 66.7  73.5 | 0.608, χ2 = 0.263 |
| I have a certified Doc  Yes  No | 33.3  22.5 | 66.7  77.5 | 0.383, χ2 = 0.760 |
| Quarantine Protocol  Yes  No | 20.4  47.1 | 79.6  52.9 | 0. 034, χ2 = 4.520* |
| Cleaning and disinfection Protocol  Always/frequently  Rarely/never | 20.9  42.9 | 79.1  57.1 | 0. 067, χ2 = 3.356* |
| Dispose waste by burning or bury  Yes  No | 14.3  28.8 | 85.7  71.2 | 0. 414, χ2 = 0.666 |
| Dispose waste by selling as fertilizer  Yes  No | 30.0  25.0 | 70.0  75.0 | 0.675, χ2 = 0.176 |
| Dispose waste as open dump  Yes  No | 25.0  30.0 | 75.0  70,0 | 0.675, χ2) = 0.176 |

*Variables significant at p ≤ 0.250 and forwarded for multivariate analysis
